# HIV-1 integrase inhibitors are substrates for the multidrug transporter MDR1-P-glycoprotein

**DOI:** 10.1186/1742-4690-4-17

**Published:** 2007-03-07

**Authors:** Maurizio Cianfriglia, Maria Luisa Dupuis, Agnese Molinari, Antonio Verdoliva, Roberta Costi, Clementina Maria Galluzzo, Mauro Andreotti, Andrea Cara, Roberto Di Santo, Lucia Palmisano

**Affiliations:** 1 Department of Drug Research and Evaluation, Istituto Superiore di Sanità, Viale Regina Elena 299, 00161 Rome, Italy; 2 Tecnogen SCpA, Piana di Monte Verna, Caserta, Italy; 3 Istituto Pasteur-Fondazione Cenci Bolognetti, Dipartimento Di Studi Farmaceutici, P.le Aldo Moro 5, 00185 Rome, Italy

## Abstract

**Background:**

The discovery of diketoacid-containing derivatives as inhibitors of HIV-1 Integrase (IN) (IN inhibitors, IINs) has played a major role in validating this enzyme as an important target for antiretroviral therapy. Since the *in vivo *efficacy depends on access of these drugs to intracellular sites where HIV-1 replicates, we determined whether the IINs are recognized by the multidrug transporter MDR1-P-glycoprotein (P-gp) thereby reducing their intracellular accumulation. To address the effect of IINs on drug transport, nine quinolonyl diketo acid (DKA) derivatives active on the HIV-1 IN strand transfer (ST) step and with EC50 ranging from 1.83 to >50 μm in cell-based assays were tested for their *in vitro *interaction with P-gp in the CEM-MDR cell system. IINs were investigated for the inhibition and induction of the P-gp function and expression as well as for multidrug resistance (MDR) reversing ability.

**Results:**

The HIV-1 IINs act as genuine P-gp substrates by inhibiting doxorubicin efflux and inducing P-gp functional conformation changes as evaluated by the modulation of UIC2 mAb epitope. Further, IINs chemosensitize MDR cells to vinblastine and induce P-gp expression in drug sensitive revertants of CEM-MDR cells.

**Conclusion:**

To our knowledge, this is the first demonstration that HIV-1 IINs are P-gp substrates. This biological property may influence the absorption, distribution and elimination of these novels anti HIV-1 compounds.

## Background

The emergence of HIV-1 strains resistant to reverse transcriptase and protease inhibitors and the toxicity associated to the chronic use of antiretroviral agents highlights the need to develop antiviral compounds with novel mechanisms of action [1].

The virally encoded integrase (IN) protein is an essential enzyme in the life cycle of the HIV-1 virus and represents an attractive and validated target for the development of antiretroviral agents [2]. Drugs that selectively inhibit this enzyme (IN inhibitors, IINs), when used alone and in combination regimens, have shown potent anti-HIV activity and a good safety profile in phase II clinical trials conducted in treatment-naïve and treatment-experienced HIV+ patients [3-5]

Drug disposition and interaction are important components of the activity and response to antiretroviral drugs. Determinants of drug disposition include the ATP binding cassette (ABC) drug transporter proteins [6]. In particular, considerable attention is now given to understanding the role of the multidrug transporter MDR1-P-glycoprotein (P-gp) in modulating drug bioavailability in cells and tissues [7].

P-gp, which is encoded in humans by the multidrug resistance (MDR) gene 1 (*mdr1*), is a membrane phosphoglycoprotein that functions as an ATP-dependent drug efflux system for structurally different compounds [8,9]. P-gp was initially studied in the setting of anticancer treatment and was identified as the agent removing a number of drugs from the cells, resulting in what has been termed MDR in tumor cells [10-13]. Concerning HIV-1 infection, it has been recently shown that MDR1-P-gp binds and removes from the drug-treated cells several HIV-1 protease inhibitors (PIs), including the recently approved Atazanavir [8,14-18].

P-gp is naturally present in CD4+ lymphocytes [19-21], one of the main cell targets of HIV-1, and in the endothelial cells lining the small blood capillaries of blood-brain, blood-testis and blood-nerve barriers, preventing the entry of toxic compounds under physiological conditions in potential HIV-1 sanctuary sites in the body [22-24]. The oral bioavailability of drugs and their penetration into the foetus also appear to be hindered by P-gp activity [25]. These findings indicate that P-gp plays an important role in the pharmacokinetic of anti-HIV-1 compounds; however, the inhibition of P-gp induced by different agents or by the combination of anti-HIV-1 drugs themselves may affect the efficacy and penetration of other anti-HIV-1 compounds [8].

On the basis of these considerations, it appears that the effect on MDR1-P-gp expression is an important component of the preclinical evaluation of new antiretroviral compounds, especially IINs, which are among the most promising new anti-HIV-1 agents [26], currently in phase III of clinical development. This study was designed to investigate, by a variety of assays, interactions between IINs and P-gp, potentially influencing their pharmacological activity.

## Results and Discussion

### Antiviral activity of IINs

Nine in house synthesized IINs [27], selected for their inhibitory activity on the stand transfer (ST) step of HIV-1 integration, were assessed for anti-HIV-1 activity and cytotoxicity on HIV-infected H9 target cells. The results are summarized in Table 1, and show that all tested IINs act as efficient enzyme inhibitors. Three of them (RDS 1974, RDS 1981 and RDS 2022) possessed a relatively low cytotoxicity but exerted a weak antiviral activity (EC50 > 50 μM) in the cell based assay, whereas the RDS 1983, RDS 1984, RDS 1992, RDS 1997 and RDS 2012 exerted a good antiviral activity associated to a relatively low cytotoxicity. In contrast, the good antiviral activity of the RDS 1996 was associated with a relatively high cytoxicity that discouraged its further development as an anti HIV-1 compound.

**Table 1:** Inhibition of integration strand transfer, anti-HIV activity and cytotoxicity in the HIV infected H9 cell line of the tested HIV-1 integrase inhibitors.

Compound (DKA derivatives)	Strand Transfer IC50* (μM)	Anti-HIV activity EC50§ (μM)	Cytotoxicity CC50^ (μM)
RDS 1974	32	>50	>50
RDS 1981	0.45	>50	>50
RDS 1983	0.25	5.98	>50
RDS 1984	0.019	9.64	>50
RDS 1992	0.70	20.5	>50
RDS 1996	0.34	24.79	2.80
RDS 1997	0.012	2.44	>50
RDS 2012	0.54	1.83	>50
RDS 2022	0.042	>50	>50

* 50% Inhibitory Concentration; § 50% Effective Concentration; ^ 50% Cytotoxic Concentration

### IINs induce a functional P-gp conformation

**Figure 1:**
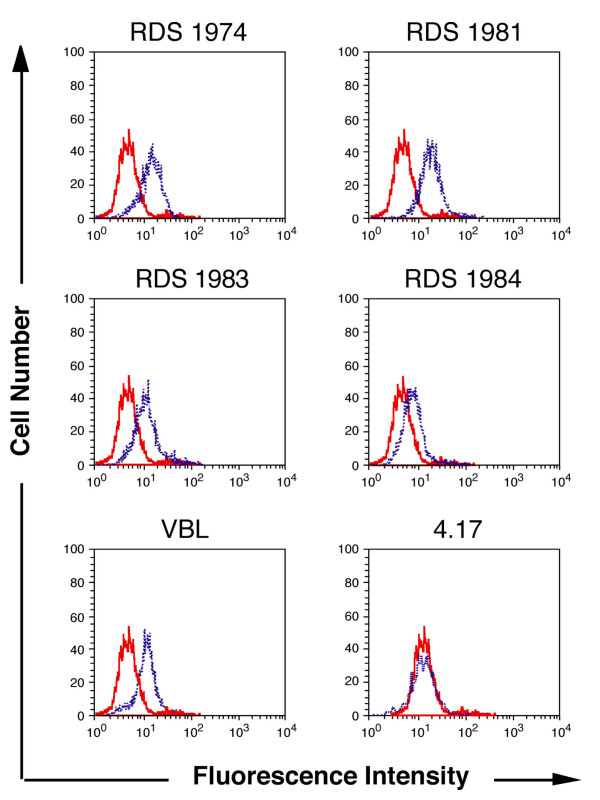
**Functional conformation of P-gp induced by IINs**. Fluorescence profile of mAb UIC2 staining on MDR CEM-VBL10 cells incubated in presence of the drug diluent (red histogram), 50 μg/ml of the indicated IINs (blue histogram), or the P-gp substrate vinblastine (10 μg/ml, VBL). MAb MM4.17 staining was carried out in identical conditions in MDR CEM-VBL10 cells incubated with drug diluent (red histogram) or 50 μg/ml of the RDS1974 (blue histogram).

Previous studies demonstrated that the reactivity of the mAb (monoclonal antibody) UIC2 with cells expressing P-gp on their surface is increased at 37°C in the presence of P-gp transport substrates or agents inducing ATP depletion [28-30]. We therefore analyzed the effect of the IINs on UIC2 epitope modulation (Fig 1 and Table 2). To better appreciate this phenomenon the CEM-VBL10 cell line was used as MDR1 cell substrate because of the relatively low number of P-gp binding sites/cell (< 1 × 10^4^) [31-33]. In general, cell lines with a higher level of P-gp require higher concentrations of P-gp substrates for maximal stimulation. As shown in Fig. 1, mAb UIC2 reactivity was increased in the presence of 50 μg/ml each of RDS 1974, RDS 1981, RDS 1983, RDS 1984 and 10 μg/ml vinblastine (VBL), a conventional P-gp substrate, while the binding level of the anti-P-gp mAb, MM4.17 was not modified (Fig. 1). This latter mAb recognizes an extracellular epitope of the human P-gp [30], but does not show the same P-gp ability of mAb UIC2 in intercepting the P-gp modulation during drug transport activity [34]. Importantly, the IINs did not modulate other cell surface antigens such as CD4 (data not shown).

**Table 2:** Induction of functional conformation and drug transport inhibition exerted by other IINs in CEM MDR cells.

Compound	Concentration μg/ml	UIC2 epitope up-modulation (A)	Doxorubicin efflux inhibition (B)
RDS 1992	25	NT	+
	50	+	++
	100	++	NT
RDS 1996	25	NT	+
	50	NT	++
	100	NT	NT
RDS 1997	25	NT	+
	50	+	++
	100	++	NT
RDS 2012	25	NT	+
	50	+	++
	100	++	NT
RDS 2022	25	NT	+
	50	+	++
	100	++	NT

A, modulation of UIC2 epitope was studied in CEM-VBL10 cells; B, the effect of IINs on the doxorubicin transport inhibition was analysed in CEM-VBL100 cells; NT, not tested; the + or ++ symbols indicate arbitrary units in measuring and differentiating the level of UIC2 epitope modulation and doxorubicin retention.

### Induction of P-gp expression by the IINs in CD4+ CEM cells

Similarly to some of the isozymes involved in drug metabolism, the expression of P-gp is inducible [35]. It is therefore important to assess whether P-gp induction occurs upon exposure to IINs, with potential implications for drug metabolism. To this aim, we investigated whether the treatment with the IINs RDS 1974, RDS 1983, RDS 1984 and RDS 1996, all having different biological and physicochemical properties (Table 1), modulate P-gp expression in the human CD4+ CEM cell system. The parental drug sensitive CEM cell line and the revertant of its derivative CEM-VBL10 MDR cell variant (CEMrev), expressing very low or undetectable amount of P-gp [33], were cultured in the presence of the indicated IINs (10 μg/ml for RDS 1974 and RDS 1996; 25 μg/ml for RDS 1983 and RDS 1984) for 28 days (RDS 1983, RDS 1984 and RDS 1996) or 104 days (RDS 1974). As a control for P-gp modulation, CEMrev cells were exposed to VBL, shown to increase P-gp expression.

Flow cytometry studies (Table 3) showed that, compared with the drug diluent control, exposure to IINs jinduced P-gp over-expression in the CEMrev cell line; the magnitude of this effect depended on treatment duration and relative drug cytotoxicity. In particular, cells that were treated for a longer period of time showed a higher percentage of P-gp positive cells. As expected, VBL exerted a strong induction of P-gp expression in CEMrev cell line. Conversely, no increase in P-gp expression was seen upon exposure of the parental drug sensitive CEM cells to the same IINs. Moreover, IINs treatment and P-gp induction were not associated with modulation of the level of CD4 expression, which was monitored throughout IINs treatment (data not shown). The observation that the IINs RDS 1974, RDS 1983, RDS 1984 and RDS 1996 modulate P-gp expression only in cells having an up-regulation of the *mdr1 *gene in their *in vitro *cell culture background [34], suggests that the induction of P-gp by IINs treatment in normal human cells is an unlikely event. Consequently, one may reasonably rule out that these IINs induce P-gp expression in T-lymphocytes of HIV-1 infected patients, leading to reduced antiviral activity of IINs and other P-gp substrates. However, the complexity of the cellular mechanisms involved in the selection of MDR variants has only partially been investigated in this study and it is well known that the MDR phenotype is multifactorial [35]; therefore it cannot be excluded *a priori *that, under IINs selective pressure, other ABC transporters may be modulated.

**Table 3:** Induction of P-gp expression in CEM and CEMrev cell lines exposed to IINs

Compound	Concentration	Days of culture	% of P-gp expressing cells	
			CEM	CEMrev

None			0	1–3
RDS 1974	10 μg/ml	104	0	25–30
RDS 1983	25 μg/ml	28	0	7–10
RDS 1984	25 μg/ml	28	0	15–18
RDS 1996	10 μg/ml	28	0	17–20
Vinblastine	10 ng/ml	28	ND*	30–35
		104	ND*	60–70

ND, not done

* % of P-gp expressing cells could not be evaluated due to VBL toxicity. Prolonged and stepwise VBL treatment induced high percentage of P-gp expressing cells [31].

### P-gp drug efflux is affected by IINs

**Figure 2:**
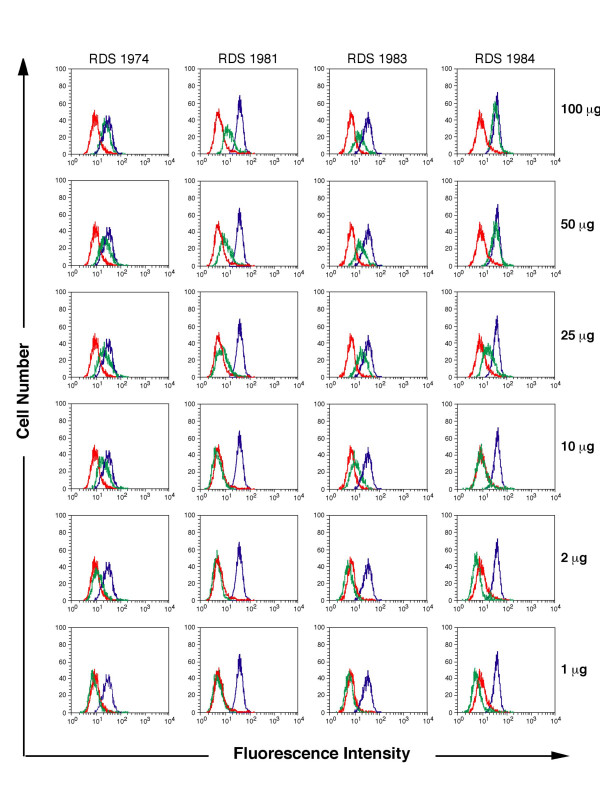
**Drug transport inhibition mediated by IINs**. Evaluation of efflux of the dye P-gp substrate doxorubicin in CEM-VBL100 MDR cells. Efflux was monitored in drug-free conditions (red histogram), in the presence of the potent P-gp blocker Verapamil (2.5 μg/ml) (blue histogram) or following incubation with several IINs (RDS1974, RDS1981, RDS1983 and RDS1984) (green histogram) at the indicated concentrations (range 1 μg/ml to 100 μg/ml).

**Figure 3:**
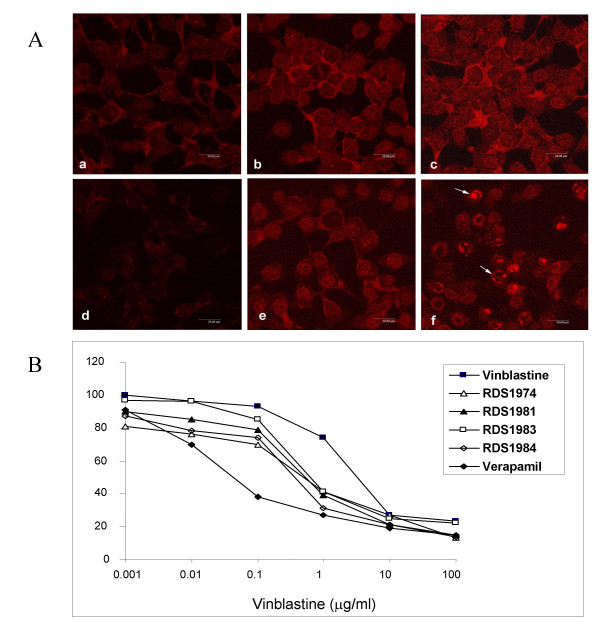
**P-gp inhibition and MDR chemosensitization**. In **(A)**, KB-V1 MDR cells were incubated at 37°C for 1 hr with 5 μg/ml doxorubicin alone or in presence of 2.5 μg/ml verapamil or 25 μg/ml RDS 1984. After washing the cells were reincubated again in identical conditions and doxorubicin efflux/retention were analysed in confocal microscopy after 1 h (panel a-c) and 3 hrs (panel d-f). The natural efflux of doxorubicin P-gp mediated is shown in panel a and d, while the doxorubicin retention due to the P-gp drug transport inhibition exerted by verapamil and RDS 1984 is shown in panel b-c (1 h incubation) or e-f (3 hrs incubation). In **(B)**, dose-response cytotoxicity to vinblastine in CEM-VBL100 MDR cells in presence of verapamil (2.5 μg/ml) or 10 μg/ml of the IINs RDS 1974, RDS 1981, RDS 1983 and RDS 1984 is shown. The values (formazan absorbance at 440 nm in ELISA reader) were calculated as % of control cells cultured in presence of IINs only or verapamil. The mean of triplicate measurements is shown; the SD was < 15% of each single value.

Doxorubucin is a fluorescent substrate for P-gp and incubation of P-gp-positive cells with this drug, followed by washing and further incubation at 37°C, results in a diminished fluorescence profile due to the active drug transport exerted by the efflux system. The presence of P-gp inhibitors such as verapamil during incubation and/or drug extrusion, restores doxorubicin fluorescence. As shown in Fig. 2 the IINs RDS 1974, RDS 1981, RDS 1983 and RDS 1984 are all capable of causing an intracellular accumulation of doxorubicin with a dose dependent effect in CEM-VBL100 MDR cells. The P-gp inhibition exerted by IINs was also investigated in an independent MDR+ cell system. Figure 3 (A) reports the results of confocal microscopy studies showing that KB-V1 P-gp -overexpressing cells at physiological conditions eliminate the dye P-gp substrate doxorubicin from the cells, and a weak or absent fluorescent signal is observed after 1 and 3 hrs of drug extrusion (Fig 3, panels a and d). In contrast, in the presence of verapamil or RDS 1984, the doxorubicin was retained in MDR KB-V1 cells and the drug was detected in cell membranes, nuclei and in marginalized chromatin (Fig 3, panels b-c and e-f). To investigate the potential use of IINs as chemosensitizing MDR agents, their effect in potentiating VBL cytotoxicity in CEM-VBL100 MDR cells was analysed (Fig 3B). Interestingly, all the tested IINs lowered the VBL resistance profile indicating a significant inhibition of P-gp function. In these experiments the IINs exerted a lower MDR reversing ability than verapamil, a drug that has been tested in clinical trials to chemosensitize MDR tumours [36]; nevertheless, in view of their relatively low *in vitro *cytotoxicity, this class of IINs should be further investigated as potential chemosensitizing compounds for P-gp expressing MDR tumours.

## Conclusion

IINs are among the most promising agents for the treatment of HIV infection [26], with two of them being in an advanced stage of clinical development [3-5]. To our knowledge, this is the first study showing that IINs are substrates for P-gp. However, we do not know whether this property is shared by all IINs or is restricted to the diketo acid class of IINs tested in this study [27], acting as P-gp substrates by inducing the up-modulation of UIC2 epitope and inhibiting doxorubicin efflux in MDR CEM-VBL100 cells (Table 2 and Fig 2). Concerning the compounds that are under clinical trials, while the MK 0518 [3,4] is a chemically distinct compound, the GS-9137 [5] is a quinolonyl diketoacid derivative comparable to the molecules used in our study. Thus, with good approximation, for the GS-9137 we may hypothesize a similar pattern of response as that observed for the DKA used in the study. Further studies aimed at evaluating the interaction of other clinically significant IINs with the P-gp system will be needed to better address this question.

In our opinion, a successful P-gp modulation may add further interest to this highly promising class of antiretroviral agents and open new perspectives for their clinical use in fields other than HIV infection.

## Materials and methods

### Integrase Inhibitors and Chemicals

Nine quinolonyl diketoacid derivatives inhibiting the strand transfer step of HIV-1 integration (IC50: 0.042–32 μM) [27] were used in this study. Verapamil (Isoptin) was provided by BASF-Knoll (Milan, Italy), Vinblastine (Velbe) by Eli Lilly (Paris, France) and Doxorubicin by Farmitalia (Nerviano, Italy).

### Antiviral activity and cytotoxicity

Anti-HIV activity was measured in the human T lymphoid H9 cells. To this purpose, cells were cultured in RPMI 1640, supplemented with 2 mM L-glutamine, penicillin, streptomycin and 10% fetal bovine serum (FBS), and infected with the HTLVIIIB laboratory strain of HIV-1 virus (100000 TCID_50 _– Tissue Culture Infective Dose- per 10^6 ^PBMC). After two hours of incubation, cells were washed with medium, and cultured at 37°C (5000 cells/well in 96-well microplates) for 3 days in presence of medium and test compounds at concentrations ranging from 50 μM to 0.1 μM. After 3 days p24 antigen concentration in the supernatants was measured by an ELISA assay (Innotest HIV antigen mAb, Innogenetics NV Belgium). Cell viability was determined by the trypan blue exclusion method.

The 50% inhibitory drug concentration (IC50) and the 50% cytotoxic drug concentration (CC50) were calculated by the median effect equation using Calcusyn Version 2.0 program (Biosoft Cambridge)

### Cell lines

The multidrug resistant (MDR) variants CEM-VBL10 and CEM-VBL100 cells were isolated by stepwise selection of the parental drug sensitive CCRF-CEM (CEM) in the presence of increasing concentrations of VBL [31,32]. Cells were grown under standard conditions for mammalian cells cultured in suspension. The basic medium (BM) for cell culturing consisted of RPMI-1640 supplemented with 10% foetal calf serum (FCS), L-glutamine (2 mM) penicillin (100 U/mL) and streptomycin (100 U/mL). All these components were purchased from Hyclone (Logan, Utah). Identical BM, culture conditions and trypsin (Hyclone) were used for the adherent MDR variant KB.V1 of the human oral epidermoid carcinoma KB cells [10]. To test the ability of selected IINs (RDS 1974, RDS 1981, RDS 1984 and RDS 1996) to induce *de novo *expression of P-glycoprotein, the drug-sensitive CEMrev cell line was used; this cell line derives from the CEM-VBL10 MDR cell line, cultured for more than 2 years in VBL-free medium and expresses very low (1–3%) or undetectable amounts of P-gp [32].

### MDR efflux assay

CEM-VBL100 cells (1 × 10^6^) were loaded with doxorubicin (10 μg/ml) in 1 ml of BM in the presence of a several IINs (concentrations ranging from 50 μg/ml to 1 mg/ml) or Verapamil (2.5 μg/ml) for 1 h at 37°C. The cells were incubated with doxorubicin (10 μg/ml) only or drug diluent in parallel cultures. At the end of incubation, the cells were washed in serum-free medium and resuspended in BM in the presence of the IINs or Verapamil (drug diluent was added in control samples) for a further 1 h at 37°C. Finally, cells were washed twice with ice-cold PBS/FACS, and analyzed in a flow cytometer (FACScan, Becton Dickinson, San Josè, CA).

### Monoclonal antibodies and UIC-2 Shift assay

The anti CD4-FITC mAb was purchased from Vinci Biochem, Firenze, Italy. The mAb UIC2 [30] was kindly provided by Dr. E. Mechetner (Chemicon Inc, Temecula, CA). For determination of P-gp expression, the mAb MM4.17, recognizing an extracellular P-gp epitope on intact/living human MDR cells [28], was also used. Both UIC2 and MM4.17 mAbs were used in a highly purified form.

The UIC2 shift assay was performed under physiological conditions as previously described [29,33]. CEM-VBL10 cells (1 × 10^6^) were resuspended in 1 ml of PBS containing 2% FCS and allowed to equilibrate at 37°C in a water bath for 10 min. The various IINs were added to samples (final concentrations 100 or 50 μg/ml) and incubated for additional 15 min at 37°C with purified UIC2 mAb (final concentration 12.5 μg/ml). VBL (10 μg/ml), which is a well known UIC2 shifting agent, and the drug diluents were used as positive and negative controls, respectively. Cells were then washed twice in ice-cold PBS containing 2% FCS with 0.01 % sodium azide (Shift Stop Buffer, SSB), stained on ice in SSB for additional 15 min with 5 μg/ml of fluorescein -conjugated goat-antimouse antibody (FITC-GAM, Cappel, West Chester, Pa, USA), washed twice with ice cold PBS/FACS and maintained in ice until flow cytometry analysis. The UIC2 shift is the difference between UIC2 binding in the presence versus the absence of the IINs under physiological conditions (37°C).

### Flow cytometry and confocal microscopy

For confocal laser-scanning microscopy (CLSM) analyses, KB-V1 adherent cells which express high level of MDR1 P-glycoprotein [10] were grown in WillCo-dishes (WillCo Wells B.V., Amsterdam, The Netherlands) for 24 hours. For P-gp inhibition experiments, the cells were incubated with 5 μg/ml doxorubicin for 1 h at 37°C in presence and absence of the IIN RDS 1984 (25 μg/ml). After washing, the cells were incubated for further 1 and 3 hrs at 37°C in the same above described conditions to allow the efflux/block of doxorubicin. CSLM observations were performed using a Leica TCS 4D apparatus (Leica Lasertechnik GmbH, Heidelberg, Germany), equipped with an argon-krypton laser, 488 nm-dichroic splitter and LP515 long pass filter. Image acquisition and processing were conducted using the SCANware (Leica) and Adobe Photoshop (Adobe Systems Inc., Mountain View, CA) software programs.

### MDR reversing

For the evaluation of the MDR reversing ability, CEM-VBL100 cells in exponential phase of growth were collected, extensively washed with warm RPMI-1640 and resuspended at the concentration of 5 × 10^3 ^cells/ml in BM alone, or in the presence of the IINs or Verapamil, as appropriated. Then cells were seeded (in triplicate) in 96-wells Costar plates (Costar, Rochester, NY) in which different VBL concentrations were previously added. Within its inhibitory range, the drug decreased growth of all cell lines proportionally to drug concentration. Cell proliferation was determined by adding 10 μg/well of PreMix WST-1 (PreMix WST-1 cell proliferation kit, Vinci Biochem, Firenze, Italy) to the cultures and measuring the absorbance at about 440 nm in a microplate ELISA reader after 4 hrs incubation (48 hrs in total) [37]. The relative cell growth was calculated by applying the formula (En-E0)/(Cn-C0) where E0 and En are the initial and after 48-treatment absorbance values in the drug-containing cultures, and C0 and Cn are the corresponding absorbance values in the untreated control culture. The obtained dose-response profile fulfilled the concentration inhibiting growth by 50% (IC50).

## Competing interests

The author(s) declare that they have no competing interests.

## Authors' contributions

**CM **conceived and planned the biological approach of this study, participated in the design and coordination of the research and drafted the manuscript.

**DML **conceived and conducted all the cell biological experiments to demonstrate the P-gp substrate activity of the IINs.

**MA **carried out confocal microscopy studies for the visualization of P-gp mediated activity of the IINs

**VA **purified and characterized at the biochemical level all the mAbs used in this study and utilized for cell line phenotyping

**CR **collaborated in the design and synthesis of Integrase Inhibitors

**GCM and AM **carried out the studies on antiviral activity and cytotoxicity of the IINs in cell based assays.

**CA **was involved in revising the manuscript critically

**DSR **conceived and designed the IINs

**PL **coordinated and supervised the study, interpreted the results and participated in drafting the manuscript
